# Clinicopathological significance and prognosis of long noncoding RNA SNHG16 expression in human cancers: a meta-analysis

**DOI:** 10.1186/s12885-020-07149-w

**Published:** 2020-07-16

**Authors:** Ruonan Jiao, Wei Jiang, Xin Wei, Mengpei Zhang, Si Zhao, Guangming Huang

**Affiliations:** Medical Center for Digestive Diseases, the Second Affiliated Hospital of Nanjing Medical University, Nanjing, 210011 China

**Keywords:** Long noncoding RNA; SNHG16, Cancer, Prognosis, Meta-analysis

## Abstract

**Background:**

Recent studies have highlighted the important role of long non-coding RNA SNHG16 in various human cancers. Here, we conducted a meta-analysis to investigate the effect of SNHG16 expression on clinicopathological features and prognosis in patients with different kinds of human cancers.

**Methods:**

We performed a systematic search in electronic databases including PubMed, EMBASE, Cochrane Library and Web of Science, to investigate the potential association between SNHG16 expression and prognostic significance and clinical features in cancer patients. Odds ratios (ORs) or hazards ratios (HRs) with corresponding 95% confidence intervals (95% CIs) were pooled to estimate the prognosis value of SNHG16 by StataSE 15.0 software.

**Results:**

A total of 16 eligible studies with 1299 patients were enrolled in our meta-analysis. The results revealed that increased expression level of SNHG16 was significantly associated with larger tumor size (OR: 3.357; 95% CI: 2.173–5.185; *P* < 0.001), advanced TNM stage (OR: 2.930; 95% CI: 1.522–5.640; *P* = 0.001) and poor histological grade (OR: 3.943; 95% CI: 1.955–7.952; *P* < 0.001), but not correlated with smoking status (*P* = 0.489), sex (*P* = 0.932), distant metastasis (*P* = 0.052), or lymph node metastasis (*P* = 0.155). Moreover, the pooled HR showed that elevated expression SNHG16 was associated with a significantly poorer overall survival (OS) (HR = 1.866, 95% CI: 1.571–2.216, *P* < 0.001). For the set of cancer types, high expression of SNHG16 was significantly associated with shorter OS in patients with cancers of the urinary system (HR: 2.523, 95% CI:1.540–4.133; P <0.001), digestive system (HR: 2.406, 95% CI:1.556–3.721; P <0.001), and other cancers (including glioma and non-small cell lung cancer) (HR: 1.786, 95% CI:1.406–2.267; P <0.001).

**Conclusions:**

LncRNA SNHG16 overexpression might serve as an unfavorable prognostic factor, which provides a basis for medical workers to evaluate the prognosis of patients and to help the decision-making process.

## Background

Cancer is a major disease that greatly endangers human health across the world. There were an estimated 14.1 million new cancer cases and 8.2 million cancer deaths globally in 2012 [1]. The incidence of cancer is increasing due to the growth and aging of the population, the intensification of industrialization and urbanization, and lifestyle modifications [1]. Thus, the burden of cancer cannot be ignored.

Mounting evidence has documented that dysregulation of tumor-suppressor genes and oncogenes is associated with human cancers [2]. However, little is known about the molecular and genetic mechanisms of tumors. Therefore, it is urgent to identify novel biomarkers for predicting the prognosis of patients with different types of cancer, which will improve their survival outcomes.

Long non-coding RNAs (lncRNAs) are composed of more than 200 nucleotides, but they do not encode proteins because they lack a recognizable open reading frame [3]. LncRNAs serve as guides, enhancers, scaffolds, or decoys by interacting with themselves or other signals in different pivotal physiological or pathological processes [4, 5]. Recent studies have demonstrated that deregulated expression of lncRNAs plays an important role in cancer development and progression, and in the recurrence, metastasis, invasion, and growth of tumors [6–8]. Thus lncRNAs can be regarded as promising biomarkers for prognosis in various types of cancers.

Small nucleolar RNA host gene 16 (*SNHG16*) is a recently discovered lncRNA [9]. Recent studies have highlighted the important prognostic role of *SNHG16* in various types of cancer, including bladder cancer [10, 11], cervical cancer [12], colorectal cancer [13], esophageal squamous cell carcinoma [14], gastric cancer [15], glioma [16], hepatocellular carcinoma [17–19], non-small cell lung cancer [20], osteosarcoma [21, 22], ovarian cancer [23], pancreatic cancer [24], and papillary thyroid cancer [25]. Some studies have revealed that upregulated *SNHG16* expression predicted poor prognosis for some cancers [26]. But some studies reported that overexpressing SNHG16 have tumor suppressing effect in some cancers, including hepatocellular carcinoma and acute lymphoblastic leukemia [27, 28]. Moreover, the expression level of *SNHG16* is closely related to TNM stage, tumor size, histological grade, overall survival (OS), and other clinical attributes [17]. And SNHG16 participates in regulating the biological functions of tumor cells through complex regulatory mechanisms, such as cell proliferation, migration, invasion and apoptosis [29]. Therefore, we conducted a meta-analysis to investigate whether the lncRNA *SNHG16* can be used as a prognostic biomarker for human cancers.

## Methods

### Search strategies

Electronic databases including PubMed, EMBASE, Cochrane Library, and Web of Science were searched. The search time was from the establishment of each database to June 20, 2019. The literature search terms included “Small nucleolar RNA host gene 16” or “SNHG16” or “Long non coding RNA SNHG16,” and “cancer” or “carcinoma” or “tumor” or “neoplasm.” The references of relevant literature were tracked for additional relevant studies.

### Literature inclusion and exclusion criteria

After the literature search, two researchers independently assessed the literature. The inclusion and exclusion criteria are displayed in Table 1.

**Table 1:** Literature inclusion and exclusion criteria

Selection criteria	
Inclusion	
(1) Topic of study: human cancer	
(2) Diagnosis method: pathology or histology	
(3) Detected method of SNHG16: qRT-PCR, ISH, or other methods in tissues	
(4) Patients divided into “high SNHG16” and “low SNHG16” groups	
(5) Association between SNHG16 and clinicopathological and prognostic features^a^: clearly reported	
(6) HR and 95% CIs: acquired or estimated	
Exclusion	
(1) Literature type: reviews, case reports, meeting abstracts, and basic experimental research literature	
(2) Duplicate articles or data	
(3) Publication language: other than English	

Abbreviations: *OS* overall survival, *qRT-PCR* quantitative reverse transcription polymerase chain reaction, *ISH* in situ hybridization, *HR* hazard ratio, *95% CI* 95% confidence interval

^a^ smoking status, sex, distant metastasis, lymph node metastasis, tumor number, tumor size, TNM stage, histological grade, and OS

### Data extraction and quality assessment

We recorded the following information: first author, publication date, country, cancer type, number of patients, sample type, sample detection method, cut-off value of *SNHG16* expression level, clinical features mentioned above, HR and 95% CI of OS. If HR and 95% CI were provided in the study, we extracted them directly. If the relevant data were not reported, we extracted and analyzed data from Kaplan-Meier curves for OS according to the method described by Tierney [30]. Two investigators independently assessed the data, and when there were differences, a third researcher decided whether or not to include the study. Two researchers independently used the Newcastle-Ottawa Scale (NOS) to evaluate the quality of the included studies. Literature with a score ≥ 6 were defined as high quality.

### Statistical analysis

Meta-analysis was performed with StataSE15.0 (Stata Corporation). Heterogeneity tests were performed based on Cochran’s *Q* and Chi-square-based *I*^*2*^ tests. If *P* > 0.10, *I*^*2*^ < 50% indicates that there is no significant heterogeneity in each study, and statistical analysis was performed using a fixed effects model; otherwise there was significant heterogeneity between the studies and a random effects model was used for the analysis. Subgroup analysis was used to explore sources of heterogeneity. The odds ratio (OR) and 95% CIs were combined to assess the association of *SNHG16* expression with clinicopathological parameters, and the HR and 95% CI included in each study were combined to map the forest to evaluate the effect of *SNHG16* expression on OS in human cancers. Publication bias was quantified using Begg’s funnel plot and Egger’s test. The reliability of the meta-analysis was tested by a sensitivity analysis. *P* < 0.05 was considered statistically significant.

## Results

### Data selection and basic characteristics

**Fig. 1:**
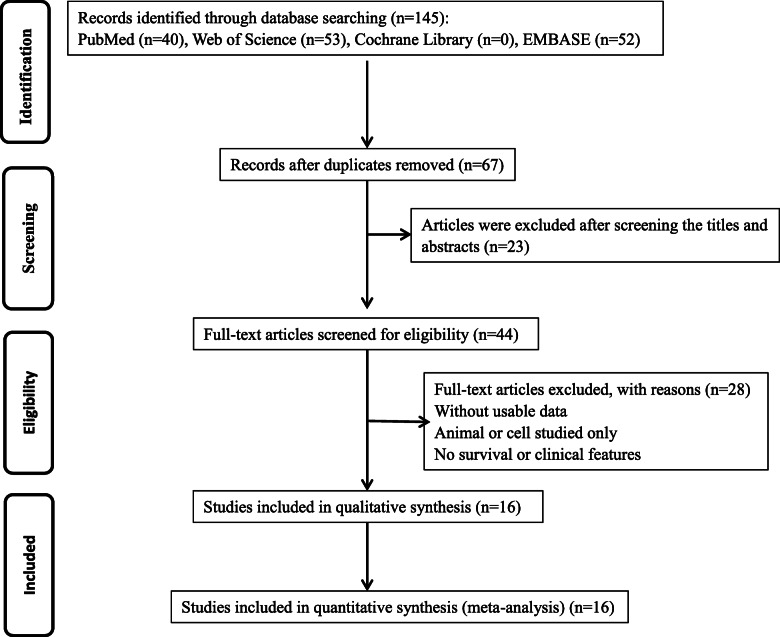
Flow chart of the literature search and selection process

A total of 145 articles were retrieved (PubMed (*n* = 40), EMBASE (*n* = 52), Cochrane Library (*n* = 0), and Web of Science (*n* = 53)). According to the above-mentioned literature inclusion and exclusion criteria, 16 articles [10–25], consisting of 1299 patients, were finally included. The number of patients in the included studies ranged from 32 to 275 patients. All the research studies were from China. Twelve types of human cancers were included in the meta-analysis, including bladder cancer, cervical cancer, colorectal cancer, esophageal squamous cell carcinoma, gastric cancer, glioma, hepatocellular carcinoma, non-small cell lung cancer, osteosarcoma, ovarian cancer, pancreatic cancer, and papillary thyroid cancer. The expression level of *SNHG16* was detected by using qRT-PCR in fifteen studies, and only one study used ISH. OS was reported in fourteen studies, and disease free survival (DFS) and progression free survival (PFS) were reported in only one study. Thus, OS was selected as the major survival outcome for our meta-analysis. HR was extracted directly in five studies and estimated from survival curves indirectly in the other 9 studies. The cut-off values for the expression level of *SNGH16* were different in these studies, including the mean, median, and fold change compared with non-tumor tissues, and in the study using ISH, strongly positive samples were defined as having high expression of *SNGH16*. The summary of screening results of the literature is shown in Table 2, and a flow chart describing the literature search and selection process is provided in Fig. 1.

**Table 2:** Characteristics of included studies

Study (year)	Country	No. of patient	Cancer type	Sample	Method	Cut-off	Outcome	Extract method	NOS score
Cao (2018) [10]	China	46	Bladder cancer	Tissue	qRT-PCR	Mean	OS	Survival curves	8
Peng (2019) [11]	China	275	Bladder cancer	Tissue	qRT-PCR	Mean	OS	Data in paper	8
Zhu (2018) [12]	China	38	Cervical cancer	Tissue	qRT-PCR	–	OS	Survival curves	6
Li (2019) [13, 26]	China	56	Colorectal cancer	Tissue	qRT-PCR	Median	OS	Survival curves	8
Han (2018) [14]	China	128	Esophageal squamous cell carcinoma	Tissue	qRT-PCR	Median	OS	Data in paper	8
Wang (2019) [15, 22]	China	32	Gastric cancer	Tissue	qRT-PCR	Median	OS	Survival curves	8
Lu (2018) [16]	China	48	Glioma	Tissue	qRT-PCR	Median	OS PFS	Data in paper	7
Ye (2019) [17]	China	103	Hepatocellular carcinoma	Tissue	qRT-PCR	Mean	–	–	6
Guo (2019) [18]	China	61	Hepatocellular carcinoma	Tissue	ISH	–	OS	Data in paper	6
Lin (2019) [19]	China	88	Hepatocellular carcinoma	Tissue	qRT-PCR	Mean	OS	Survival curves	8
Han (2018) [14]	China	66	Non-small cell lung cancer	Tissue	qRT-PCR	Median	OS DFS	Data in paper	8
Liao (2019) [21]	China	96	Osteosarcoma	Tissue	qRT-PCR	Mean	OS	Survival curves	7
Wang (2019) [15, 22]	China	65	Osteosarcoma	Tissue	qRT-PCR	Median	OS	Survival curves	7
Yang (2018) [23]	China	103	Ovarian cancer	Tissue	qRT-PCR	–	OS	Survival curves	6
Liu (2019) [24]	China	46	Pancreatic cancer	Tissue	qRT-PCR	Median	OS	Survival curves	8
Wen (2019) [25]	China	48	Papillary thyroid cancer	Tissue	qRT-PCR	–	–	–	6

Abbreviations: *OS* overall survival, *PFS* progression free survival, *DFS* disease free survival, — not available, *qRT-PCR* quantitative reverse transcription polymerase chain reaction, *ISH* in situ hybridization, *NOS* Newcastle–Ottawa Scale

### The association between SNHG16 expression and clinicalpathological features

To demonstrate the clinical features of *SNHG16* expression level in human cancers, we analyzed and summarized all the clinicopathological data from the included studies. As shown in Table 3, five studies composed of 373 patients revealed a significant association between *SNGH16* overexpression and larger tumor size (OR: 3.357; 95% CI: 2.173–5.185; *P* < 0.001) using a fixed effects model, and no heterogeneity was found (*I*^*2*^ = 0%; *P* = 0.813). In eight studies including 591 patients, we found that overexpression of *SNGH16* had a significant correlation with advanced TNM stage (OR: 2.930; 95% CI: 1.522–5.640; *P* = 0.001). A random effects model was performed for the analysis of TNM stage because of the heterogeneity (*I*^*2*^ = 64.200%; *P* = 0.007). A total of three studies including 187 patients reporting the relationship of *SNGH16* expression with histological grade were analyzed. Our data demonstrated that elevated *SNGH16* expression was associated with poor histological grade (OR: 3.943; 95% CI: 1.955–7.952; *P* < 0.001). Due to no heterogeneity (*I*^*2*^ = 13.800%; *P* = 0.313), a fixed effects model was used. However, no significant relationship between *SNHG16* expression and smoking status, sex, distant metastasis and lymph node metastasis was found in the meta-analysis.

**Table 3:** Meta-analysis of the studies reporting the association between over-expressed SNHG16 and clinicopathological parameters

Clinicopathological parameters	Studies	Patients	Model	OR (95% CI)	*P* value	Heterogeneity		
						I^2^(%)	χ^2^	*P*-value
Smoking (yes vs no)	4	296	Fixed	1.175 (0.744–1.854)	0.489	8.3	3.27	0.351
Sex (male vs female)	12	1051	Fixed	1.286 (0.766–1.277)	0.932	0.0	5.05	0.929
Distant metastasis (yes vs no)	5	362	Random	3.033 (0.991–9.281)	0.052	78.8	18.89	0.001
Lymph node metastasis (yes vs no)	9	777	Random	1.923 (0.781–4.735)	0.155	83.8	49.38	0.000
Tumor number (multiple vs single)	2	378	Fixed	0.829 (0.531–1.293)	0.409	0.0	0.01	0.910
Tumor size (≥5 cm vs<5 cm)	5	373	Fixed	3.357 (2.173–5.185)	0	0.0	1.57	0.813
TNM stage (III/IV vs I/II)	8	591	Random	2.930 (1.522–5.640)	0.001	64.2	19.58	0.007
Histological grade (poorly vs well/moderately)	3	187	Fixed	3.943 (1.955–7.952)	0	13.8	2.32	0.313

Abbreviations: *OR* odd ratio, *95% CI* 95% confidence interval

### The association between SNHG16 expression and overall survival

**Fig. 2:**
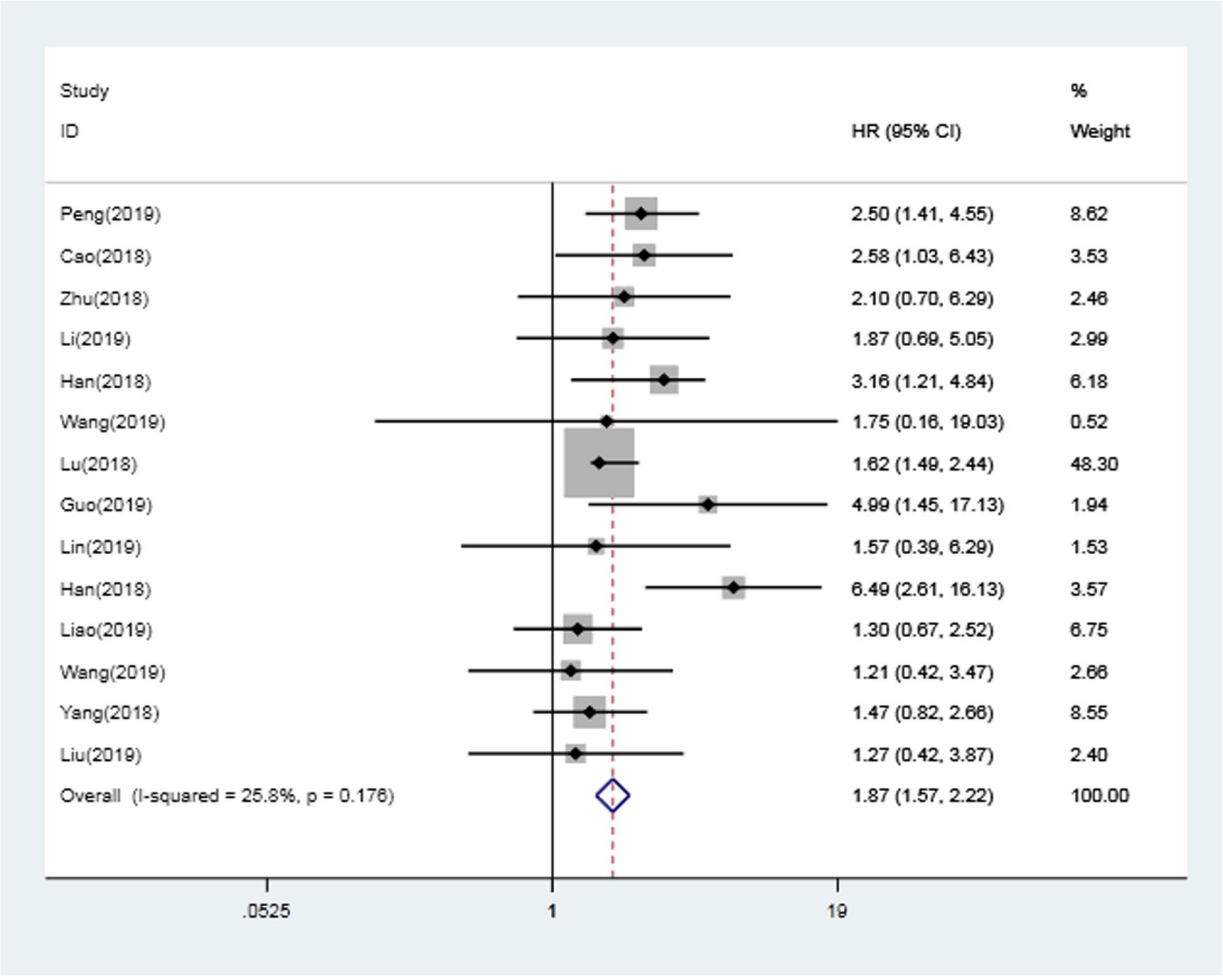
Forest plot for the relationships between lncRNA *SNHG16* expression and OS

As presented in Table 4 and Fig. 2, in total, 14 articles reporting the association between *SNHG16* expression level and OS, including 1148 patients, were included in the meta-analysis. The results showed that high *SNHG16* expression was significantly correlated with poor OS (HR: 1.866; 95% CI: 1.571–2.216; *P* <0.001). There was no heterogeneity (*I*^*2*^ = 25.800%; *P* = 0.176) in the data, so a fixed effects model was used. In addition, subgroup analysis for extract method and detection method was performed. The subgroup analysis revealed that the extract method of HR, either the data in paper or survival curves, had a significant influence on OS (data in paper: HR: 2.912; 95% CI: 1.729–4.906; *P* < 0.001; survival curves: HR: 1.571; 95% CI: 1.155–2.135; *P* = 0.004), and the heterogeneity results were *I*^*2*^ = 13.500%, *P* = 0.009, *I*^*2*^ = 2.260%, *P* = 0.972, respectively. For the detection method of *SNHG16* expression, the overall HR for the qRT-PCR group for OS was 1.830 (95% CI:1.538–2.177, *P* < 0.001), with no heterogeneity (*I*^*2*^ = 20.200%, *P* = 0.239). Compared with the group with a low expression level of *SNHG16*, upregulated *SNHG16* showed a statistically significant decrease in OS. For the set of cancer types, high expression of SNHG16 was significantly associated with shorter OS in patients with cancers of the urinary system (HR: 2.523, 95% CI:1.540–4.133; P <0.001), digestive system (HR: 2.406, 95% CI:1.556–3.721; P <0.001), and other cancers (including glioma and non-small cell lung cancer) (HR: 1.786, 95% CI:1.406–2.267; P <0.001). However, in terms of the reproductive system and musculoskeletal system, elevated SNHG16 expression was not predictive of unfavorable OS (HR = 1.592, 95% CI: 0.948–2.674, *P* = 0.079; HR:1.274, 95% CI: 0.727–2.233, *P* = 0.398, respectively). Besides other cancers, all of the above cancers showed little heterogeneity between them (I^2^ < 50%, *P* > 0.1). For other cancers, significant heterogeneity was found (I^2^ = 87.9%, *P* = 0.004) (Table 3), which may be due to the differences between cancers of different systems.

**Table 4:** Overall and subgroup analysis of SNHG16 for OS in human cancers

Variables	Studies	Patients	Model	HR (95% CI)	*P*-value	Heterogeneity		
						I^2^(%)	χ2	*P*-value
OS	14	1148	Fixed	1.866 (1.571–2.216)	0.000	25.8	17.52	0.176
Extract method								
Data in paper	5	578	Random	2.912 (1.729–4.906)	0.000	70.40	13.5	0.009
Survival curves	9	570	Fixed	1.571 (1.155–2.135)	0.004	0.00	2.26	0.972
Method								
qRT-PCR	13	1087	Fixed	1.830 (1.538–2.177)	0.000	20.2	15.04	0.239
ISH	1	61	–	4.985 (1.451–17.129)	0.011	–	–	–
Cancer type								
Urinary System	2	321	Fixed	2.523 (1.540–4.133)	0.000	0.0	0.0	0.955
Digestive System	6	411	Fixed	2.406 (1.556–3.721)	0.000	0.0	3.89	0.566
Reproductive system	2	141	Fixed	1.592 (0.948–2.674)	0.079	0.0	0.32	0.575
Musculoskeletal system	2	161	Fixed	1.274 (0.727–2.233)	0.398	0.0	0.01	0.910
Other	2	114	Fixed	1.786 (1.406–2.267)	0.000	87.9	8.30	0.004

Abbreviations: *HR* hazard ratio, *95% CI* 95% confidence interval, *OS* overall survival, *qRT-PCR* quantitative reverse transcription polymerase chain reaction, *ISH* in situ hybridization

### The association between SNHG16 expression and disease free survival / progression free survival

As presented in Table 5, there is just one study providing data on DFS or PFS respectively. We couldn’t make meta-analysis to pool the results. As shown in Table 5, Han et al. reported that high SNHG16 expression was significantly correlated with poor DFS (HR: 4.505; 95% CI: 1.980–10.309; P <0.001). Lu et al. demonstrated that high SNHG16 expression was related with shorter PFS (HR:3.167; 95% CI:1.552–6.231; P<0.021).

**Table 5:** The association between SNHG16 expression and DFS/PFS

Study (year)	No. of patient	Cancer type	Outcome	HR (95% CI)	P
Lu (2018) [16]	48	Glioma	PFS	3.167 (1.552–6.231)	0.021
Han (2018) [14]	66	Non-small cell lung cancer	DFS	4.505 (1.980–10.309)	<0.001

Abbreviations: *PFS* progression free survival, *DFS* disease free survival, *HR* hazard ratio, *95% CI* 95% confidence interval

### Sensitivity analysis

**Fig. 3:**
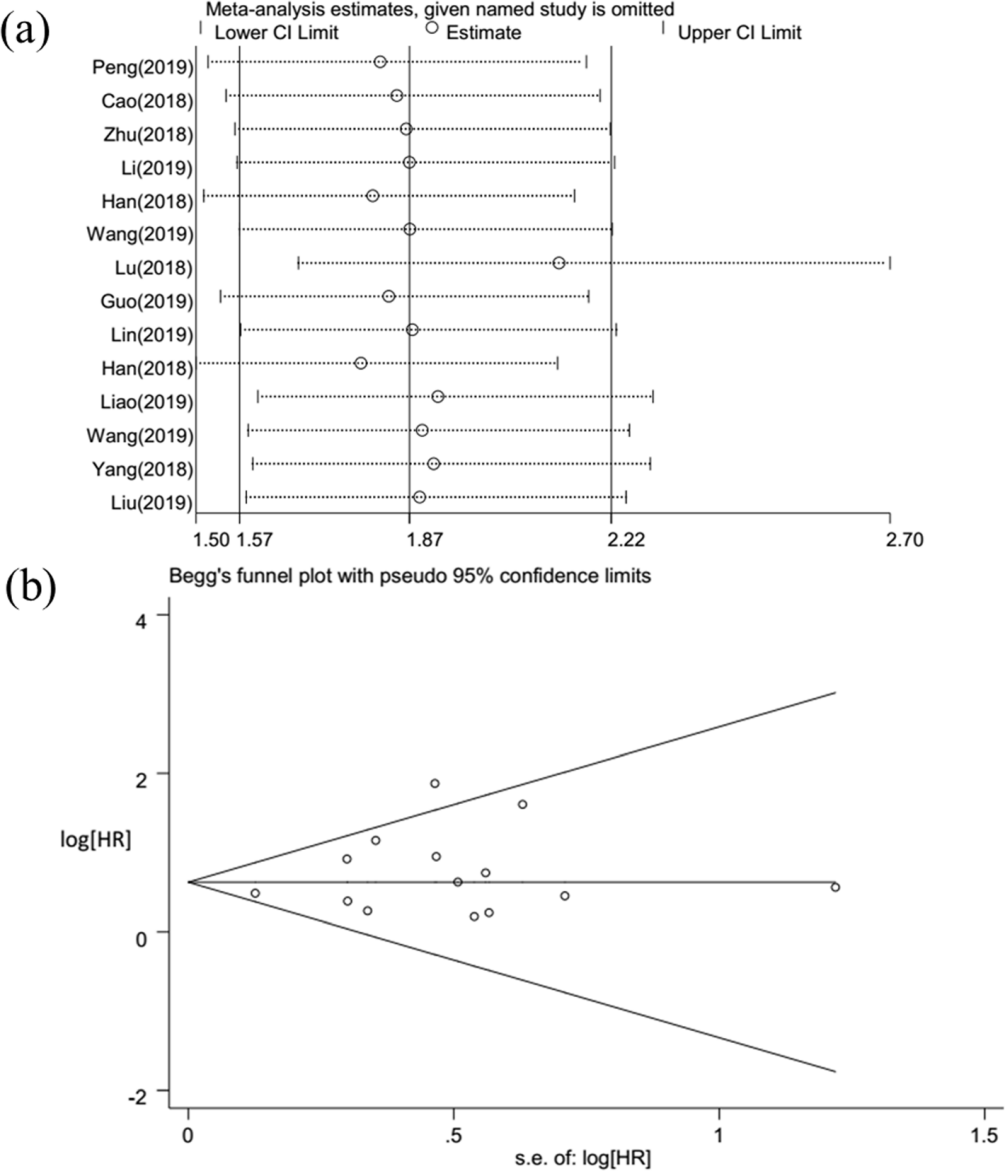
Sensitivity analysis and publication bias for meta-analysis of *SNHG16* and OS. **a** Sensitivity analysis for meta-analysis of *SNHG16* and OS. **b** Funnel plot of the publication bias for OS

To identify whether individual studies had an impact on OS, sensitivity analysis was performed. The results suggested that no single study affected the stability of the HR values, indicating that the results of this meta-analysis data are stable and reliable (Fig. 3a).

### Publication bias

The potential publication bias of the meta-analysis was assessed by Begg’s funnel plot and Egger’s test. We observed that the shape of the funnel diagram was almost symmetrical and did not show any signs of significant asymmetry (Fig. 3b). As shown in Fig. 3b, there was no obvious publication bias for OS, as a result of the Begg’ test (*P* = 0.584) and Egger’s test (*P* = 0.234) (Table 6). Likewise, there was no obvious evidence for significant publication bias in terms of sex, lymph node metastasis, or TNM stage (Table 6). We did not evaluate the publication bias for smoking, distant metastasis, tumor number, tumor size, and histological grade because the number of included studies was small.

**Table 6:** Publication bias of clinicopathological parameters by Begg’s test and Egger’s test

Clinicopathological parameters	Begg’s test (*P*)	Egger’s test (*P*)
OS	0.584	0.234
Smoking (yes vs no)	–	–
Sex (male vs female)	0.115	0.14
Distant metastasis (yes vs no)	–	–
Lymph node metastasis (yes vs no)	0.754	0.738
Tumor number (multiple vs single)	–	–
Tumor size (≥5 cm vs<5 cm)	–	–
TNM stage (III/IV vs I/II)	0.711	0.604
Histological grade (poorly vs well/moderately)	–	–

Abbreviations: *OS* overall survival

## Discussion

Many studies have found that lncRNAs play a crucial role in human cancers and inflammatory diseases by regulating different levels of gene expression programs, such as transcription, post-transcriptional processes, and epigenetics [31, 32].

LncRNAs are involved in various cellular events and act as guides, signals, decoys, and dynamic scaffolds by modulating cancer hallmarks, including DNA damage, metastasis, immune escape, cell stemness, drug resistance, metabolic reprogramming, and angiogenesis [33]. LncRNAs contribute to epigenetic changes where lncRNAs have the potential to act as oncogenes and/or tumor suppressors [29]. Thus lncRNAs take an important part in cancer development and growth. And the expression or functional abnormalities of lncRNA has been identified to be associated with tumor occurrence, metastasis, progression and prognosis [34–36]. LncRNAs in general are thought to be promising as independent biomarkers for prognosis in human cancers [33].

The lncRNA *SNHG16* has been reported as a modulator in multiple cancers. Research conducted by Cao et al. indicated that *SNHG16* predicted poor prognosis, which can promote tumor proliferation by epigenetically silencing p21 in bladder cancer [10]. Meanwhile, *SNHG16* contributes to sorafenib resistance by sponging miR-140-5p in hepatocellular carcinoma [17]. Christensen et al. found that *SNHG16* was upregulated in colorectal cancer by affecting lipid metabolism [9]. Lian et al. reported that the expression of *SNHG16* was significantly associated with invasion depth, lymph node metastasis, TNM stage, and histological differentiation in gastric cancer [37]. Several studies have shown that patients with elevated expression of *SNHG16* had poor OS in comparison with those with low levels [10–16, 18–24]. Not only in cancer, recent evidence suggest that *SNHG16* also has a significant impact on regulating the inflammatory response. For example, *SNHG16* can regulate LPS-induced inflammation injury in WI-38 cells by targeting miR-146a-5p/CCL5 [38].

This meta-analysis aimed to investigate the relationship between the expression level of *SNHG16* and the pathological features in different types of human cancers. A total of 1299 patients from 16 studies were included. The fixed or random effect model was used for evaluating the smoking status, sex, distant metastasis, lymph node metastasis, tumor number, tumor size, TNM stage, and histological grade. We found that a high expression level of *SNHG16* was correlated with larger tumor size, poor histological grade, and advanced TNM stage. Although elevated *SNHG16* expression was associated with smoking status, high proportion of male, distant metastasis, and lymph node metastasis, there was no significant correlation. Furthermore, in terms of survival outcomes, patients with high expression of *SNHG16* had significantly shorter OS than those with low *SNHG16* expression.

When the association between lncRNA SNHG16 and tumor type was explored, we found that there was a significant relationship between SNHG16 overexpression and poor OS in patients with digestive system cancers, urinary system cancers, and other system cancers (including glioma and non-small cell lung cancer). However, regarding the reproductive system cancers and musculoskeletal system cancers, elevated SNHG16 expression was not predictive of unfavorable OS. In recent years, many studies have demonstrated that abnormal expression of SNHG16 does not only correspond to one tumor, but also can been detected different tumor tissues from various systems [39–41]. And the mechanisms of SNHG16 in different tumor types are unclear and controversial [29]. Results from this meta-analysis indicated that overexpression of the lncRNA SNHG16 might serve as a prognostic factor in patients with digestive system cancers, urinary system cancers, and other system cancers (including glioma and non-small cell lung cancer), which could provide a basis for medical workers to evaluate the prognosis of patients and to help the decision-making process.

There were limitations in this study: (1) All the included studies were from China, and the included literature was only published in English. The included literature may not be enough, there may be potential publication bias; (2) The number of patients and the number of studies in some analysis groups were relatively small, and not all types of human cancers were included; (3) The cut-off value for distinguishing high or low *SNHG16* expression levels was not standard across all studies; (4) The detection method of *SNHG16* expression was different among included studies, although most of them used qRT-PCR; (5) Not all the included studies reported the HRs and their 95% CI directly, so we estimated them from survival curves, which may not be precise enough; and (6) The response to treatment of various cancer patients and the patients’ different lifestyles may also underlie some of the heterogeneity.

## Conclusion

Overexpression of the lncRNA *SNHG16* might serve as a prognostic factor, which provides a basis for medical workers to evaluate the prognosis of patients and to help the decision-making process. However, this meta-analysis has some limitations. In the future, multi-center, large-scale, and more comprehensive experimental research is still needed to verify the results of this meta-analysis.

## Data Availability

All data are included in this article.

## Abbreviations

HR: Hazard ratio
95% CI: 95% confidence interval
qRT-PCR: Quantitative reverse transcription PCR
ISH: In situ hybridization
—: Not available
OS: Overall survival
